# Dysbiosis in the Salivary Microbiome Associated with IgA Nephropathy—‍A‍ ‍Japanese Cohort Study

**DOI:** 10.1264/jsme2.ME21006

**Published:** 2021-06-01

**Authors:** Anushka Khasnobish, Lena Takayasu, Ken-ichi Watanabe, Tien Thi Thuy Nguyen, Kensuke Arakawa, Osamu Hotta, Kensuke Joh, Akiyo Nakano, Shuhei Hosomi, Masahira Hattori, Wataru Suda, Hidetoshi Morita

**Affiliations:** 1 Laboratory of Animal Applied Microbiology, Graduate School of Environmental and Life Science, Okayama University, Japan; 2 Department of Human Ecology, School of International Health, Graduate School of Medicine, The University of Tokyo, Japan; 3 Department of Otolaryngology—Head and Neck Surgery, Tohoku University Graduate School of Medicine, Sendai, Miyagi, Japan; 4 Faculty of Engineering and Technology College of Agriculture and Forestry, Hue University, Vietnam; 5 Hotta Osamu Clinic, 2–39 Rokuchonome minami-machi, Wakabayashi-ku, Sendai, Miyagi 984–0013, Japan; 6 Department of Pathology, Tohoku University Graduate School of Medicine, Sendai, Japan; 7 Department of Microbiology and Infectious Diseases, Nara Medical University, 840 Shijo-cho, Kashihara-shi, Nara 634–8521, Japan; 8 Department of Gastroenterology, Osaka City University Graduate School of Medicine, 1–4–3, Asahi-machi, Abeno-ku, Osaka, 545–8585, Japan; 9 Laboratory for Microbiome Sciences, RIKEN Center for Integrative Medical Sciences, Wako, Japan

**Keywords:** salivary microbiome, IgA nephropathy, oral microbiota, kidney disease, random forest algorithm

## Abstract

IgA nephropathy is one of the leading causes of chronic kidney disease in Japan. Since the origin and mechanisms by which IgA nephropathy develops currently remain unclear, a confirmed disease diagnosis is currently only possible by highly invasive renal biopsy. With the background of the salivary microbiome as a rich source of biomarkers for systemic diseases, we herein primarily aimed to investigate the salivary microbiome as a tool for the non-invasive diagnosis of IgA nephropathy. In a comparison of salivary microbiome profiles using 16S rRNA amplicon sequencing, significant differences were observed in microbial diversity and richness between IgA nephropathy patients and healthy controls. Furthermore, recent studies reported that patients with IgA nephropathy are more likely to develop inflammatory bowel diseases and that chronic inflammation of the tonsils triggered the recurrence of IgA nephropathy. Therefore, we compared the salivary microbiome of IgA nephropathy patients with chronic tonsillitis and ulcerative colitis patients. By combining the genera selected by the random forest algorithm, we were able to distinguish IgA nephropathy from healthy controls with an area under the curve (AUC) of 0.90, from the ulcerative colitis group with AUC of 0.88, and from the chronic tonsillitis group with AUC of 0.70. Additionally, the genus *Neisseria* was common among the selected genera that facilitated the separation of the IgA nephropathy group from healthy controls and the chronic tonsillitis group. The present results indicate the potential of the salivary microbiome as a biomarker for the non-invasive diagnosis of IgA nephropathy.

Chronic kidney disease (CKD) is a worldwide health issue that affects millions of individuals every year. According to the Japanese Society of Nephrology report in 2005, approximately 13.3 million people in Japan had CKD (Imai *et al.*, 2009). Immunoglobulin A nephropathy (IgAN) is one of the leading causes of CKD in Japan (Tomino, 2016) and is also the most common form of primary glomerulonephritis reported globally (D’Amico, 1987; Schena, 1990). With 50% of IgAN patients progressing to end-stage renal disease (ESRD) (Moriyama *et al.*, 2014), IgAN is a significant health burden, particularly in Japan, due to the rapidly aging population and annual increases in the number of patients undergoing dialysis (Masakane *et al.*, 2018). Even after more than 50 years since its first classification, a confirmed diagnosis of IgAN is only achieved by kidney biopsy (Tomino, 2016), which is a highly invasive diagnostic tool and possesses greater risk of complications, particularly in the elderly. Therefore, the development of a non-invasive diagnostic tool for IgAN is urgently needed.

IgAN is an idiopathic disease, as indicated by the multi-hit hypothesis (Suzuki *et al.*, 2011). The clinical diagnosis of IgAN includes the detection of differently glycosylated polymeric IgA1 (pIgA1) immune complex deposits in kidney glomeruli by histopathology, along with microscopic or macroscopic hematuria and proteinuria by a urinalysis (Sallustio *et al.*, 2019). However, the trigger or maintenance of these immune reactions remains unclear. Genome-wide association studies (GWAS) on large IgAN cohorts discovered loci that only account for approximately 5% of the disease risk (Sallustio *et al.*, 2019). Therefore, apart from genetics, other environmental factors (Sallustio *et al.*, 2019) are considered to be associated with the progression and pathogenesis of IgAN.

Mucosal immunity is an essential factor in the pathogenesis of IgAN because the upper respiratory tract microflora has been implicated in recurrent macroscopic hematuria (Sallustio *et al.*, 2019). The microbiota plays a vital role in the development of mucosal-associated lymphoid tissue (MALT), which, in turn, regulates the composition of the microbiota (Nakajima *et al.*, 2018; Sallustio *et al.*, 2019). IgA primarily originates from MALT, and GWAS of IgAN identified several risk loci involved in maintaining mucosal immunity (Sallustio *et al.*, 2019). The oral mucosa is a gateway to the human body, and the salivary microbiome, which typically consists of more than 200 predominant species in the oral cavity (Krishnan *et al.*, 2017), plays a crucial role in oral mucosal immunity (Moutsopoulos and Konkel, 2018). Salivary microbiome dysbiosis often reflects inflammatory responses and microbiome changes in the gut (Bajaj *et al.*, 2015; Abe *et al.*, 2018). Members of the salivary microbiome are potential diagnostic biomarkers for immunological diseases, such as rheumatoid arthritis (Chen *et al.*, 2018), primary sclerosing cholangitis (Iwasawa *et al.*, 2018), and pancreatic cancer (Torres *et al.*, 2015; Coit *et al.*, 2016). Furthermore, the collection and storage of saliva are non-invasive, and inexpensive (Hemadi *et al.*, 2017). These factors may be advantageous for conducting salivary microbial profiling in IgAN patients to identify non-invasive microbial biomarkers for effective low-risk diagnostics of IgAN.

GWAS also revealed common risk loci between IgAN and IBD, such as Crohn’s disease (CD) and ulcerative colitis (UC) (Sallustio *et al.*, 2019). A Swedish population-based study reported that patients with IgA nephropathy were more likely to develop inflammatory bowel diseases (Rehnberg *et al.*, 2021). A transgenic murine model of IgAN highlighted the essential dependence of signals from the commensal microbiota for kidney IgA deposition in the pathogenesis of IgAN (McCarthy *et al.*, 2011). Few studies have reported a change in the composition of the gut (De Angelis *et al.*, 2014; Dong *et al.*, 2020; Hu *et al.*, 2020), salivary (Piccolo *et al.*, 2015; Luan *et al.*, 2019), periodontal (Cao *et al.*, 2018), or tonsillar (Park *et al.*, 2020) microbiota in Caucasian, Chinese, and Korean IgAN patients. However, none of these studies investigated microbiome changes in IgAN versus IBD.

In Japan, tonsillectomy monotherapy or tonsillectomy paired with steroid pulse therapy is one of the most effective therapeutic regimens for early-stage primary IgAN patients (Xie *et al.*, 2003; Nihei *et al.*, 2017). The efficacy of this treatment is attributed to the relationship between focal tonsillar infection and increases in pIgA-secreting plasma cells in the tonsils of IgAN patients (Meng *et al.*, 2012). Previous studies detected pathogenic bacteria, commonly associated with chronic tonsillitis (CT) and periodontitis, in the tonsillar crypts of IgAN patients (Jensen *et al.*, 2013; Nagasawa *et al.*, 2014; Watanabe *et al.*, 2017). Despite previous findings implicating periodontal pathogens in the pathogenesis of IgAN, no studies have examined the salivary microbiome of IgAN and compared it with that of chronic tonsillitis patients.

In the present study, we performed a 16S rRNA gene sequence-based analysis of the salivary microbiome of IgAN, UC, and CT patients and healthy subjects in a Japanese cohort. In our attempt to distinguish IgAN from other mucosal diseases (UC and CT), we identified a set of potential microbial biomarkers that may differentiate IgAN from other diseases and healthy individuals. According to the Ministry of Health, Labour and Welfare 2005 survey in Japan, male sex is one of the predictive factors for IgAN (Tomino, 2016). Therefore, we herein investigated the sex-specific association of the salivary microbiome in IgAN patients.

## Materials and Methods

### Sample collection and DNA extraction

We excluded patients with any history of antimicrobial usage within the past three months and gastrointestinal or hepatobiliary surgery from the present study. Saliva samples were collected preoperatively from IgAN and CT patients. All subjects were prohibited from eating or drinking for 2 h before sample collection. Signed consent was obtained from subjects before sampling. The present study followed all relevant guidelines and institutional policies, and was approved by the Azabu University Ethics Committee (029, 14 March 2013), Osaka University Ethics Committee (2413, 29 September 2014), and RIKEN Ethics Committee (H30-4, 29 August 2019). Following the self-collection of salivary samples in test tubes, samples were transported to the laboratory within 24 h of collection. At the laboratory, samples were immediately frozen using liquid nitrogen and stored at –80°C until further analyses. The extraction of bacterial DNA from these salivary samples was performed as described previously (Morita *et al.*, 2007; Said *et al.*, 2014). Briefly, 1‍ ‍mL of saliva was centrifuged at 3,300×*g* at 4°C for 10‍ ‍mins. The resulting bacterial cell pellets were then suspended in 10‍ ‍mM Tris–HCl/10‍ ‍mM EDTA buffer and incubated with 15‍ ‍mg mL^–1^ lysozyme (Sigma-Aldrich) at 37°C for 1 h. Purified achromopeptidase (Wako Pure Chemical Industries) was added to the samples at a final concentration of 2,000 units‍ ‍mL^–1^ before they were incubated at 37°C for an additional 30‍ ‍min. We treated the suspension with 1% (w/v) sodium dodecyl sulphate (SDS) and 1‍ ‍mg mL^–1^ proteinase K (Merck Japan) and incubated samples at 55°C for 1 h. The resultant lysate was treated with phenol/chloroform/isoamyl alcohol (Life Technologies Japan) followed by centrifugation at 3,300×*g* at 4°C for 10‍ ‍min. To precipitate DNA, a 1/10 volume of 3M sodium acetate (pH=4.5) and 2 volumes of ethanol (Wako Pure Chemical Industries) were added to the supernatant. DNA pellets, obtained by centrifugation at 3,300×*g* at 4°C for 15‍ ‍min, were then rinsed with 75% ethanol, dried, and dissolved in 10‍ ‍mM Tris–HCl/1‍ ‍mM EDTA (TE) buffer. DNA samples were purified by a treatment with 1‍ ‍mg mL^–1^ RNase A (Wako Pure Chemical Industries) at 37°C for 30‍ ‍min and precipitated by adding equal volumes of 20% polyethylene glycol solution (PEG6000-2.5MNaCl). These samples were centrifuged at 8,060×*g* at 4°C and double-rinsed with 75% ethanol before pellets were dried and dissolved in TE buffer.

PCR amplification of the V1-V2 hypervariable region of the 16S rRNA gene was performed using barcoded 27Fmod (5′-AGRGTTTGATYMTGGCTCAG-3′) and 338R (5′-TGCTGCCTCCCGTAGGAGT-3′) primers (Kim *et al.*, 2013) according to a previously described protocol (Tsuda *et al.*, 2015). Following purification using AMPure XP magnetic purification beads (Beckman Coulter) and quantification using the Quant-iT PicoGreen dsDNA Assay Kit (Life Technologies Japan), equal amounts of PCR amplicons were pooled together and sequenced using the 454 GS FLX Titanium or 454 GS Junior system (Roche Applied Science) according to the manufacturer’s instructions (Said *et al.*, 2014; Tsuda *et al.*, 2015).

### Data processing of 16S rRNA sequences

After sequencing, we used an analysis pipeline designed to process the 454 pyrosequencing data of the V1-V2 region of the 16S rRNA gene, as previously reported (Kim *et al.*, 2013). Reads with an average quality score less than 25, lacking both universal primers and possible chimeric reads, accounting for 48–49% of all reads, were excluded from the analysis (Table S1). A total of 2,300 reads per sample were randomly chosen from high-quality reads for analysis in the present study. The selected reads were then sorted on the basis of the average quality score and grouped into OTUs using the UCLUST algorithm with a 96% identity threshold (Said *et al.*, 2014). Taxonomic assignments for each OTU were appointed by similarity searching using the GLSEARCH program against the RDP, CORE, and NCBI genome databases. Sequence similarity thresholds of 70, 94, and 96%, respectively, were used for these assignments at the phylum, genus, and species levels.

### Data availability

The high-quality 16S V1-V2 sequences used in the present study for a downstream analysis were deposited in the DDBJ/GenBank/EMBL database (accession no. DRA002611, DRA002617, and DRA002618 [Tsuda *et al.*, 2015], DRA011285, and DRA011286).

### Statistical analysis

Samples were sex-matched using the chi-squared test by comparing sex data across groups. Similarly, for age matching, the demographic data of samples were compared using a one-way ANOVA test. Between-sample diversity or beta diversity was assessed using the UniFrac distance metric (Lozupone *et al.*, 2011) followed by a principal coordinate analysis (PCoA) to visualize any similarities or differences in the microbiome structure of the samples. We performed a permutational multivariate analysis of variance (PERMANOVA) to assess the significance of beta diversity between the groups, and the corresponding *P* values were adjusted for multiple testing using the Benjamin-Hochberg (BH) correction method. To evaluate the alpha diversity of the samples in terms of richness and diversity, we used the observed OTU and Chao1-estimated number, and the Shannon index, respectively. The significance of relative abundance at the taxonomic levels (phylum, genus, species, and OTU) was evaluated using the Wilcoxon test with BH corrections for multiple comparisons. A similar approach was used to clarify the sex-specific microbiome association. We grouped data into male and female samples and then assessed sex effects in UniFrac weighted and unweighted metrics using PERMANOVA. The groups that showed significance (*P*<0.05, the Wilcoxon test) were then investigated further with respect to alpha and beta diversities as well as the taxonomic profiles of microbiomes specific to male and female samples in the groups.

We used a Linear Discriminate Analysis (LDA) effect size (LEfSe) tool (http://huttenhower.sph.harvard.edu/lefse/) to identify differentially abundant taxa between the IgAN, healthy, and disease (CT and UC) groups at different taxa levels (genus and OTU levels). LefSe uses the non-parametric Kruskal-Wallis test and unpaired Wilcoxon rank sum test to identify differentially abundant taxa among groups of samples and the LDA method to estimate the effect size of each feature/taxa among the groups (Segata *et al.*, 2011). In the present study, a LEfSe analysis was performed with the alpha value for statistical analyses set to 0.05 and the threshold on the logarithmic LDA score for discriminative features to 2.

We used the AUC-RF (version 1.1) (Calle *et al.*, 2011) package to generate RF models, as described in the methods by Iwasawa *et al.* (2018), to identify the group of taxa among the selected biomarkers from the LefSe analysis that may be used to classify the IgAN group from other disease and HC groups. AUCRF package-based analyses were performed using the R version 3.6.1 within the RStudio environment (version 1.2.5019).

## Results

### Study subjects, age, and sex distribution

We recruited 43 IgAN, 20 CT, and 33 UC patients and 65 healthy volunteers without any disease symptoms. We collected salivary samples from 43 patients with IgAN (the IgAN group, median age 39 years), 20 with CT (the CT group, median age 34.5), 33 with UC (the UC group, median age 47), and 65 healthy controls (the HC group, median age 37). After age and sex matching, 11 UC and 15 HC samples were removed from the present study, which changed the median age of the UC group to 44.5 and the HC group to 37.5 (Table 1).

The Ethics Committees of Azabu University, Osaka University, and RIKEN approved this study. Signed informed consent was collected from all subjects before sample collection.

### Summary of 454 reads

We obtained 1,576,683 high-quality 16S reads from the four groups using the 454 GS FLX Titanium platform (Roche Applied Science) (for details see Materials and Methods, Table S1). After removing low quality and possibly chimeric reads, we obtained 809,607 reads from 135 samples. We randomly selected 2,300 reads per sample (310,500 reads from 135 samples), and further analyzed them using the pipeline for the 454 barcoded pyrosequencing of 16S PCR amplicons to minimize overestimations of species richness in clustering due to intrinsic sequencing errors (Kim *et al.*, 2013). Good’s coverage index (Good, 1953; Singleton *et al.*, 2001) of the 2,300 reads per sample was 0.996, indicating a high degree of coverage. Therefore, sequence data were sufficient for analyses in the present study.

### Differences in alpha and beta diversities of the salivary microbiota in IgAN, CT, UC, and HC groups

**Fig. 1.**
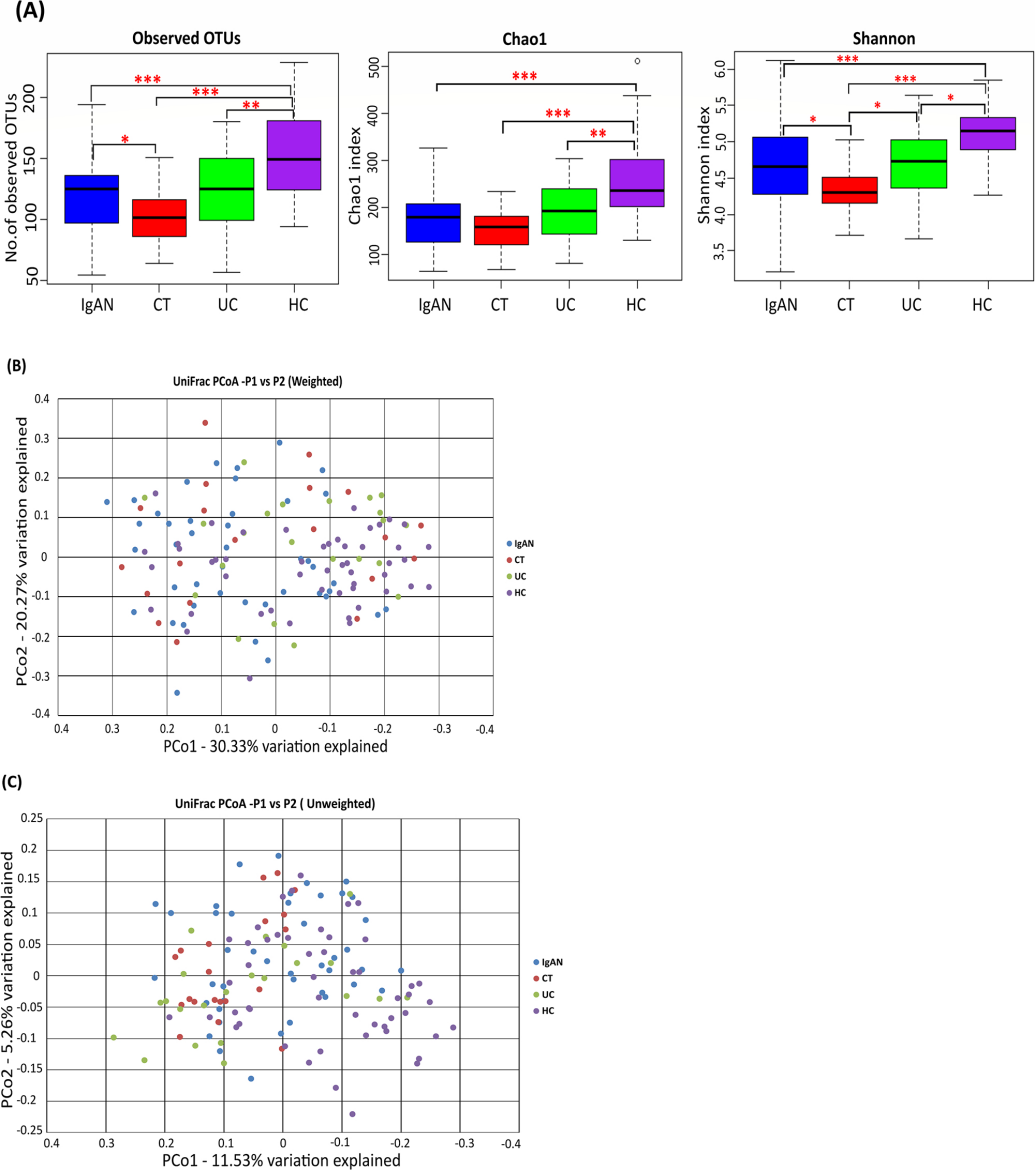
Alpha and beta diversities in IgAN, CT, UC, and HC subjects. Samples from 43 IgAN (blue), 20 CT (red), 22 UC (green), and 50 HC (purple) subjects are shown. (A) Observed and Chao1-estimated OTU numbers, and the Shannon index of the salivary microbiome from the four groups. * *P*<0.05; ** *P*<0.01; *** *P*<0.001 based on the Wilcoxon test with the Benjamin-Hochberg correction. (B) Weighted UniFrac–PCoA and (C) unweighted UniFrac–PCoA of the salivary microbiome from the four groups. OTU, operational taxonomic unit; PCoA, principal coordinate analysis; IgAN, immunoglobulin A nephropathy; CT, chronic tonsillitis; UC, ulcerative colitis; HC, healthy controls.

The observed OTU number in the IgAN group was significantly lower than that in the HC group (*P*=5.31E-05) and was significantly higher than that in the CT group (*P*=0.04407) (Fig. 1A). No significant difference was observed between OTU numbers in the IgAN and UC groups (Fig. 1A). Chao1-estimated OTU numbers were significantly higher in the HC group than in the IgAN, CT, and UC groups (*P*=1.40E-06, 6.79E-07, and 0.00459, respectively) (Fig. 1A). Within-sample diversity or alpha diversity, indicated by the Shannon index, showed a significant difference between all groups, except for between the IgAN and UC groups (Fig. 1A). The HC group had a significantly higher alpha diversity than the IgAN, CT, and UC groups (*P*=0.00048, 4.34E-08, and 0.01055, respectively), while the CT group had a significantly lower alpha diversity than the IgAN and UC groups (*P*=0.02099 and 0.01763, respectively) (Fig. 1A).

PCoA based on the unweighted UniFrac distance metric showed that many of the IgAN, CT, and UC samples were segregated from HC samples (Fig. 1C). PERMANOVA revealed a significant difference between the IgAN and CT groups in the unweighted UniFrac metric (*P*<0.01), but not in the weighted UniFrac metric (Table 2). Additionally, PERMANOVA showed that the IgAN group was significantly different from both the HC and UC groups for unweighted (*P*<0.01) and weighted (*P*<0.01) UniFrac metrics (Table 2). According to unweighted UniFrac metrics, in comparisons with the HC group, the IgAN group had lower dysbiosis than the CT and UC groups, as shown by the respective R^2^ values from PERMANOVA (R^2^ value=0.03 [IgAN vs HC]; 0.06 [CT vs HC]; 0.05 [UC vs HC]; Table 2).

### Variations in salivary microbiome taxonomic profiles between all groups

We taxonomically assigned OTUs according to phylotypes in public microbial 16S rRNA gene databases. Phyla with a relative mean abundance of more than 0.1% across all groups (IgAN, CT, UC, and HC) and, thus, accounting for 99.8% of total abundance were *Firmicutes*, *Bacteroidetes*, *Proteobacteria*, *Actinobacteria*, *Fusobacteria*, *Candidatus Saccharibacteria* (TM7), and *Streptophyta*. The relative mean abundance of *Bacteroidetes* was significantly lower (*P*=0.0014), whereas that of *Proteobacteria* was significantly higher (*P*=0.0021) in the IgAN group than in HC group. The relative mean abundance of *Actinobacteria* was significantly lower in the IgAN group than in the UC group (*P*=0.0256). *Candidatus Saccharibacteria* (TM7) was significantly more abundant in the IgAN group than in the CT group (*P*=0.0074). The ratio of the relative mean abundance of *Firmicutes*/*Bacteroidetes* was higher in the disease groups (IgAN, CT, and UC groups) than in the HC group. On the other hand, the *Firmicutes*/*Proteobacteria* ratio was lower in the disease groups than in the HC group.

The taxonomic assignment at the genus level identified 270 bacterial genera, 51 of which (Table S2) had a relative mean abundance of more than 0.1%, accounting for 97.8% of the total abundance. *Neisseria* was significantly more abundant in the IgAN group than in the HC and UC groups (*P*=0.0005 and 0.0032, respectively), whereas *Prevotella*, *Megasphaera*, and *Solobacterium* were significantly less abundant in the IgAN group than in the HC and UC groups (*P*=0.0019, 0.0335, 0.0241, 0.0261, 0.0417, and 0.0391, respectively). On the other hand, *Stomatobaculum* was significantly more abundant in the IgAN group than in the CT and UC groups (*P*=0.0385 and 0.005, respectively), but was significantly less abundant than in the HC group (*P*=0.0008). The abundance of *Peptostreptococcus* was also significantly lower in the IgAN group than in the HC group (*P*=0.0044), but higher than in the CT group (*P*=0.0431). *Peptococcus* was significantly more abundant in the IgAN group than in the CT group (*P*=0.0361). However, *Schaalia* was significantly less abundant in the IgAN group than in the CT and UC groups (*P*=0.0147 and 0.0017, respectively). *Actinomyces* and *Selenomonas* were significantly less abundant, whereas *Gemella* was significantly more abundant in the IgAN group than in the UC group (*P*=0.0020, 0.0006, and 0.0098, respectively).

Similar patterns were observed in genus abundance in comparisons of the disease groups with the HC group. The abundance of *Stomatobaculum*, *Staphylococcus*, *Cutibacterium*, and *Peptostreptococcus* was significantly lower in all disease groups (IgAN, UC, and CT) than in the HC group (*P*<0.05, Table S2). Similarly, the abundance of the genera *Veillonella*,* Solobacterium*, and *Corynebacterium* was significantly lower in the IgAN and CT groups than in the HC group (*P*<0.05, Table S2). The mean relative abundance of the genus *Enhydrobacter* was significantly lower in the IgAN and UC groups than in the HC group (*P*=0.0021 and 0.0457, respectively).

**Fig. 2.**
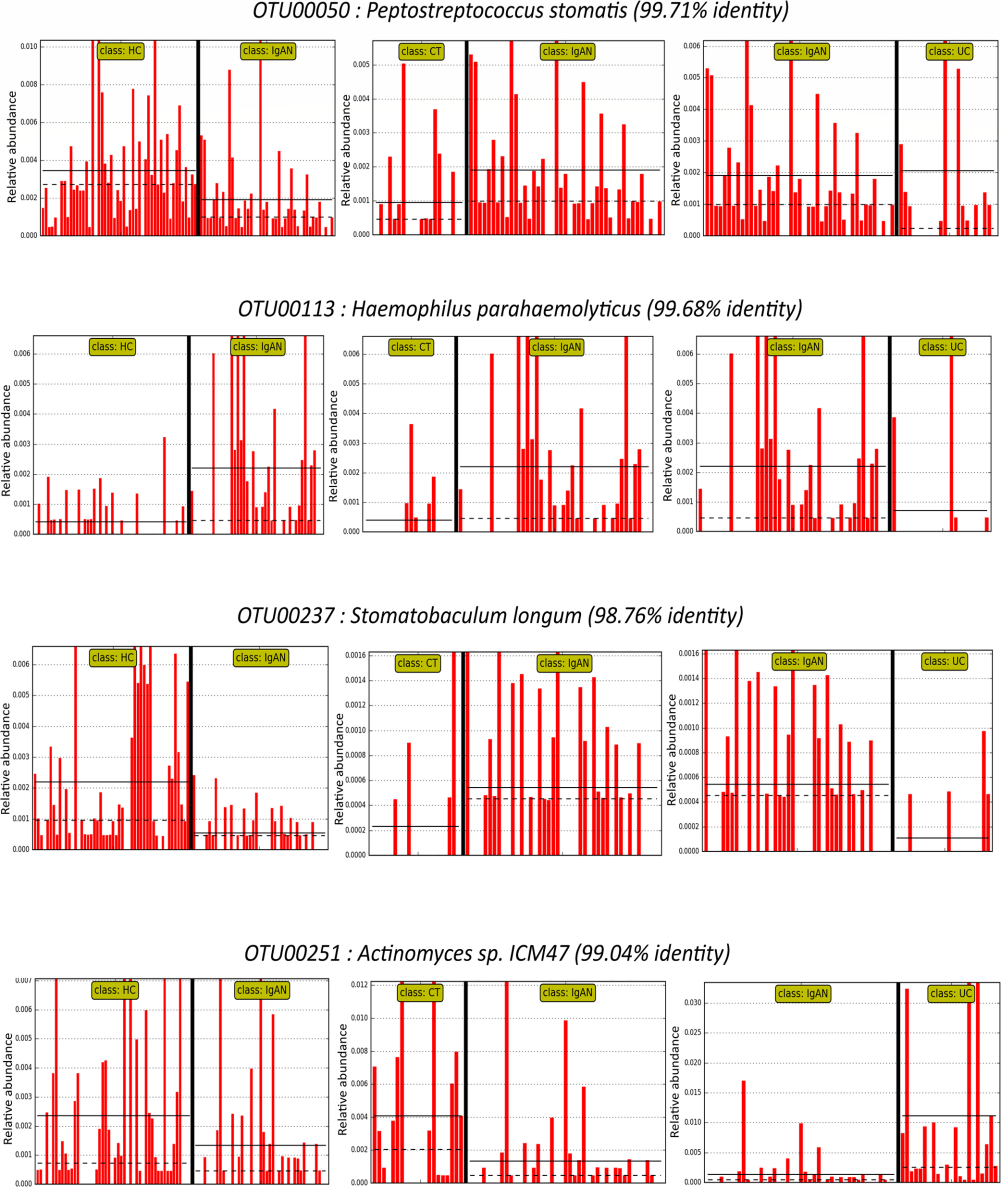
Common differential features between IgAN and three other groups (CT, UC, and HC) from a LefSe analysis. With a threshold LDA score >2, differentially abundant taxa were identified between the IgAN vs CT, IgAN vs UC, and IgAN vs HC groups. The four common taxa at the OTU level are shown. Individual red bars represent the relative abundance of the taxa in a sample. LefSe, Linear discriminant analysis (LDA) effect size; IgAN, immunoglobulin A nephropathy; CT, chronic tonsillitis; UC, ulcerative colitis; HC, healthy controls.

To further evaluate salivary microbiome differences among IgAN, CT, and UC patients and healthy subjects, the LDA LefSe method was used to identify significant discriminative features between the groups (with a logarithmic LDA score threshold >2). At the OTU level, we identified 19, 33, and 36 differential taxa between the IgAN and CT, IgAN and UC, and IgAN and HC groups, respectively (Fig. S1). Four of these taxa, namely, OTU00113 (99.68% similarity with *Haemophilus parahaemolyticus*), OTU00237 (98.76% similarity with *Stomatobaculum longum*), OTU00251 (99.04% similarity with *Actinomyces sp. ICM47*), and OTU00050 (99.71% similarity with *Peptostreptococcus stomatis*) were common differential features between the IgAN group and the other three groups (CT, UC, and HC) (Fig. 2).

**Fig. 3.**
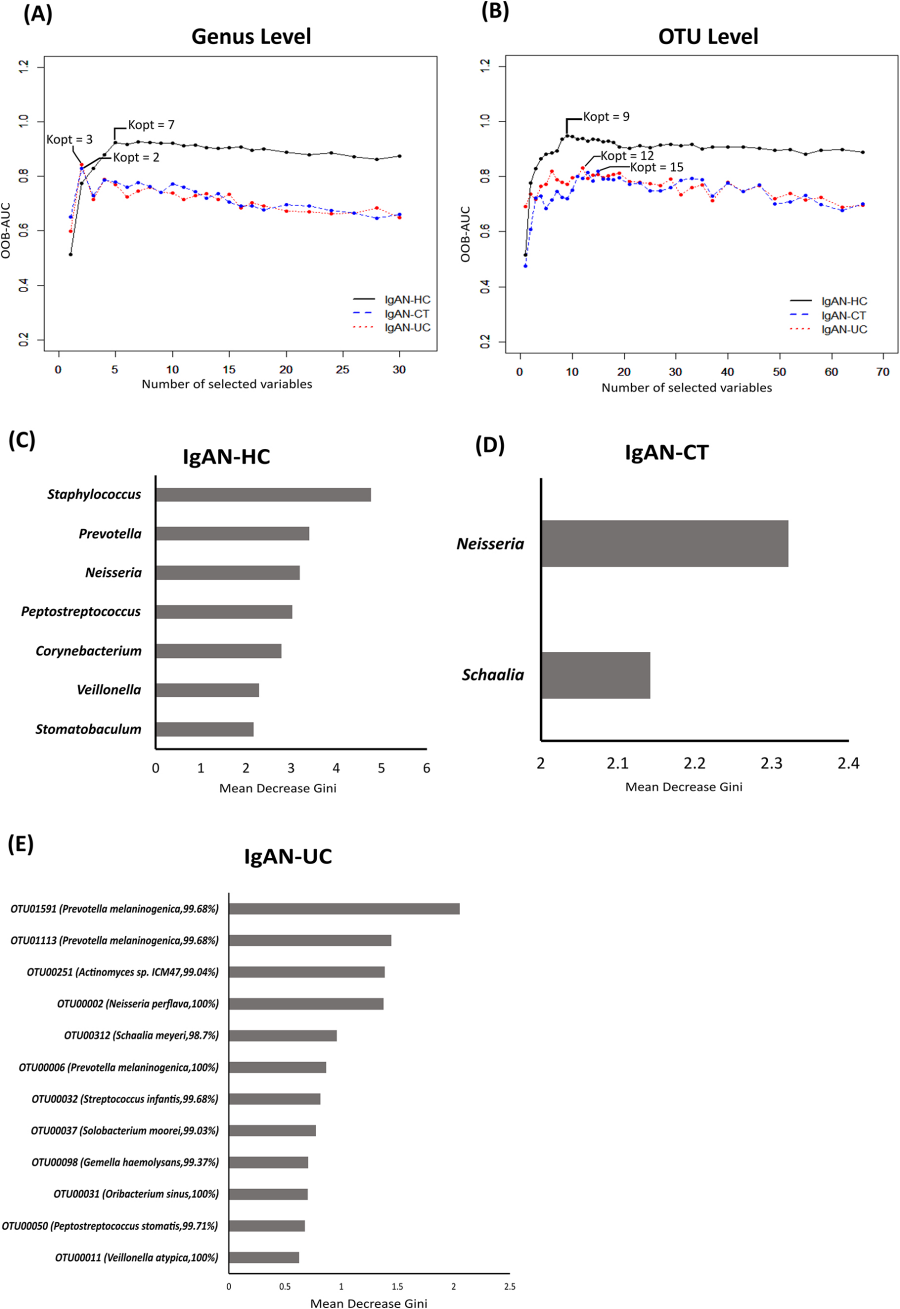
Random Forest (RF) analysis of the salivary microbiota of four groups using the AUC-RF package at the OTU level. Best RF models (comparisons based on a combination of the best mean area under the curve [AUC] value) was obtained at the (a) genus and (b) OTU levels. The selected features of the best RF models for (c) IgAN-HC, (d) IgAN-CT, and (e) IgAN-UC comparisons are shown. Kopt=optimal number of features to distinguish between two groups under comparison; IgAN, immunoglobulin A nephropathy; CT, chronic tonsillitis; UC, ulcerative colitis; HC, healthy controls.

To assess the potential value of the identified microbial biomarkers for clinically differentiating the IgAN group from the disease (CT and UC) and HC groups, we generated random forest (RF) models using the AUC-RF package. We generated models for two taxa levels (genus and OTU) (Table 3). We used the area under the curve (AUC) of the receiver operating curve (ROC), that was, in turn, based on the out-of-bag (OOB) error rate, to identify the combination of multiple taxa from the 30 differential genera selected by the LefSe tool that contributes to discriminating the IgAN group from the other groups. The models with the best AUC values at the genus level were observed for 7, 2, and 3 genera between the IgAN and HC, IgAN and CT, and IgAN and UC groups, respectively (Fig. 3A). Among the selected genera, *Neisseria* (Fig. S2C) was a common contributor for distinguishing the IgAN group from the HC, CT, and UC groups, and *Schaalia* for distinguishing the IgAN group from the CT and UC groups. The abundance of *Neisseria* was significantly higher in the IgAN group than in the HC and UC groups (*P*=0.0005 and 0.0032, respectively), whereas no significant differences were observed between the IgAN and CT groups. The abundance of *Schaalia* was significantly lower in the IgAN group than in the CT (Fig. S2B) and UC groups (*P*=0.01 and 0.002, respectively). Six‍ ‍out of the 7 genera (*Staphylococcus*, *Prevotella*, *Peptostreptococcus*, *Corynebacterium*, *Veillonella*, and *Stomatobaculum*) contributed to distinguishing the IgAN group from the HC group (Fig. S2). Among 3 genera, the genus *Selenomonas* contributed to distinguishing the IgAN group from the UC group. We confirmed these results using a 10-fold cross-validation of AUC-RF models repeated 20 times to obtain mean AUCs, which were 0.90 between the IgAN and HC groups, 0.707 between the IgAN and CT groups, and 0.851 between the IgAN and UC groups (Table 3).

At the OTU level, we used 66 differential OTUs selected by the LefSe tool to build the RF model. The models with the best AUC values had 9, 15, and 12 OTUs between the IgAN and HC, IgAN and CT, and IgAN and UC groups, respectively (Fig. 3B). Out of these OTUs, OTU00002 (100% similarity with *Neisseria perflava*) and OTU00050 (99.71% identity with *P. stomatis*) were common contributors to distinguishing the IgAN group from the HC, CT, and UC groups. The UC and HC groups had a significantly higher abundance, whereas the CT group had a significantly lower abundance of the OTU, OTU00050 (99.71% identity with *P. stomatis*) than the IgAN group (*P*=0.02, 0.01, and 0.03, respectively). OTU00002 (100% similarity with *N. perflava*) was significantly higher in abundance in the IgAN group than in the HC and UC groups (*P*=0.0002 and 0.0060, respectively); however, the difference between its abundance in the IgAN and CT groups was not significant (*P*>0.05). Four OTUS, OTU00312 (*Schaalia meyeri*, 98.7%), OTU00251 (*Actinomyces sp.* ICM47, 99.04%), OTU00011 (*Veillonella atypica*, 100%), and OTU00031 (*Oribacterium sinus*, 100%) were common contributors to distinguishing the IgAN group from the other two disease groups. OTU00312 (*S. meyeri*, 98.7% identity) was significantly lower in abundance in the IgAN group than in the CT and UC groups (*P*=0.005 and 0.005, respectively). OTU00251 (*Actinomyces sp. ICM47*, 99.04% identity) had significantly lower abundance, whereas OTU00031 (*O. sinus*, 100%) had significantly higher abundance in the IgAN group than in the UC group (*P*=0.0005 and 0.04, respectively). No significant differences were observed in the abundance of OTU00011 (*V. atypica*, 100%) between the IgAN group and the other two disease groups. The mean AUC of the 10-fold cross-validation repeated 20 times was 0.88 between the IgAN and HC groups, 0.62 between the IgAN and CT groups, and 0.88 between the IgAN and UC groups (Table 3).

### Sex-specific microbiome association

To establish whether there is any underlying sex-specific association of the salivary microbiome, we performed PERMANOVA on samples grouped according to sex. The results obtained revealed a significant difference between the male and female groups with respect to weighted and unweighted UniFrac metrics irrespective of the disease state grouping (Table S3). Upon further analyses of the dataset group-wise, a significant difference was observed between male and female samples in the IgAN group for the weighted UniFrac metric and in the HC group for the unweighted UniFrac metric only (Table S3). Observed OTU numbers were significantly lower in the IgAN male and IgAN female groups than in the HC male and HC female groups, respectively (*P*=0.0010 and 0.04, respectively) (Fig. S3A). Similarly, chao1-estimated OTU numbers were significantly lower in the IgAN male and IgAN female groups than in the HC male and HC female groups, respectively (*P*=0.0002 and 0.003, respectively) (Fig. S3A). Alpha diversity, indicated by the Shannon index, showed a significant difference between the IgAN female and HC female groups (*P*=0.005), but not between the IgAN male and HC male groups (Fig. S3A). PCoA based on the unweighted UniFrac distance metric segregated IgAN male and IgAN female samples from HC male and HC female samples, respectively (Fig. S3B). Similar sample segregation was observed between IgAN male and HC male samples and between IgAN female and HC female samples in PCoA plots based on weighted UniFrac distance metrics (Fig. S3B). A significant difference was also noted between the IgAN male and HC male groups for both weighted and unweighted UniFrac metrics (Table S3). Similar results were obtained in PERMANOVA between the IgAN female and HC female groups (Table S4).

Regarding the taxonomic composition, no phylogenetic demarcation was detected between taxa that were differentially abundant in two sex-based analyses. At the phylum level, Bacteroidetes was significantly less abundant in the IgAN male group than in the HC male group, while *Candidatus Saccharibacteria* (TM7) was significantly less abundant in the IgAN female group than in the HC female group. However, the abundance of the phylum *Proteobacteria* was significantly higher in the IgAN male and IgAN female groups than in the HC male and HC female groups, respectively. At the genus level, the abundance of 12 genera (*Neisseria*,* Rothia*, *Oribacterium*, *Turicibacter*, *Campylobacter*, *Peptostreptococcus*, *Romboutsia*, *Stomatobaculum*, *Enhydrobacter*, *Peptococcus*, *Acinetobacter*, and *Dialister*) with a mean relative abundance of more than 0.1% was significantly higher in the IgAN male group than in the HC male group. The abundance of 3 genera (*Atopobium*, *Corynebacterium*, and *Micrococcus*), with a mean relative abundance of more than 0.1%, was significantly lower, while that of the genus *Streptococcus* was significantly higher in the IgAN female group than in the HC female group (Fig. S4).

## Discussion

The human microbiome is closely associated with mucosal immunity, and previous findings implicated the gut, tonsil, periodontal, and salivary microbiota in IgAN (De Angelis *et al.*, 2014; Nagasawa *et al.*, 2014; Piccolo *et al.*, 2015; Watanabe *et al.*, 2017; Cao *et al.*, 2018; Luan *et al.*, 2019; Dong *et al.*, 2020; Hu *et al.*, 2020; Park *et al.*, 2020). However, despite Japan having the second highest incidence and frequency of IgAN (Schena and Nistor, 2018), few studies have been conducted on the IgAN-associated microbiota in a Japanese cohort. To the best of our best knowledge, the present study is the first to characterize the salivary microbiome of patients with IgAN and compare it with those of CT and UC patients and healthy subjects.

The present results revealed that the salivary microbial composition in IgAN patients was different from that in healthy subjects, as indicated by lower species richness and microbial diversity in the salivary microbiota of IgAN patients. Similar results were obtained on the salivary microbiome composition in CT and UC patients. Similarly, salivary microbial richness and diversity were previously reported to be lower (*P*>0.05) in IgAN than in HC in a Caucasian population (Piccolo *et al.*, 2015), whereas a Chinese population-based study of the IgAN salivary microbiota did not report any significant differences (Luan *et al.*, 2019). In fecal microbiome studies associated with IgAN, few studies reported lower microbial richness (De Angelis *et al.*, 2014; Hu *et al.*, 2020) and diversity (De Angelis *et al.*, 2014) in IgAN than in HC, whereas another study did not find any significant difference (Dong *et al.*, 2020). These differences may be attributed to variability in the study design, such as the use of different hypervariable regions for 16S rRNA sequencing as well as different sequencing platforms, sample sizes, and ethnicities. A statistical analysis of phylogeny-based weighted and unweighted UniFrac metrics showed that the observed dysbiosis was associated with differences in the presence or absence of the microbial taxa as well as their abundance in the population. Similar to the findings of Caucasian (De Angelis *et al.*, 2014; Piccolo *et al.*, 2015) and Chinese (Luan *et al.*, 2019; Dong *et al.*, 2020) population studies, microbial dysbiosis was detected in IgAN patients in the present study. In PERMANOVA of unweighted UniFrac distance metrics, the IgAN and CT groups significantly differed in terms of the microbial taxa composition only. In contrast, the composition and abundance of the microbial taxa contributed to a significant difference between the IgAN and UC groups. These results indicate that the overall microbial population was similar in the CT and IgAN groups. However, since this is the first study to compare the salivary microbiome of IgAN, CT, and UC patients, a more detailed and larger population-based study investigating the relationships among these diseases in terms of the microbiome is required.

Differences were observed at the taxonomic level between the IgAN group and the other groups. The phylum *Firmicutes* dominated all samples and was the most abundant in the IgAN group, which is consistent with the findings of fecal and salivary microbiota studies on IgAN patients (De Angelis *et al.*, 2014; Luan *et al.*, 2019; Hu *et al.*, 2020). The *Firmicutes*/*Proteobacteria* ratio was lower in the IgAN group than in the HC group, which was similar to that reported in a salivary microbiota study on a Caucasian population (Piccolo *et al.*, 2015) and in a periodontal microbiome study on an Asian population (Cao *et al.*, 2018), but differed from that in a salivary microbiota study on a Chinese cohort (Luan *et al.*, 2019).

The present results revealed that the *Firmicutes*/*Bacteroides* ratio was higher in the UC group than in the HC group, which is similar to the findings of other gut microbiome studies (Kabeerdoss *et al.*, 2015; Xun *et al.*, 2018). Furthermore, the abundance of the phylum *Actinobacteria* was significantly higher in the UC group than in the IgAN and HC groups, which is consistent with a meta-analysis of the gut microbiota composition in IBD (Walters *et al.*, 2014) and salivary microbiome ecotypes associated with UC (Xun *et al.*, 2018). Based on these findings, an abnormal physiological state is associated with microbial dysbiosis in gut inflammatory diseases (Xun *et al.*, 2018).

The significant differences observed between the IgAN group and the three other groups (CT, UC, and HC) in terms of the microbial taxa prompted us to investigate the capacity of these selected biomarkers to discriminate the IgAN group from the CT, UC, and HC groups. In the present study, a combination of salivary taxa separated IgAN from healthy individuals with AUC of 0.88 and 0.90 at the OTU and genus levels, respectively. A Chinese cohort study reported the separation of IgAN from HC with a predictive accuracy of up to 80% by using salivary microbial OTUs in combination with biochemical characteristics (Luan *et al.*, 2019). These findings indicate that salivary microbiome-derived biomarkers are applicable for a predictive diagnosis of IgAN from healthy population.

*Neisseria* was a common contributor for distinguishing IgAN from healthy individuals and CT. The abundance of this genus was higher in the IgAN group than in the CT, UC, and HC groups, which is consistent with the findings of a previous study (Piccolo *et al.*, 2015). Furthermore, at the OTU level, some OTUs belonging to the genus *Neisseria* were among the selected taxa for differentiating the IgAN group from the UC group and were significantly enriched in IgAN patients. Salivary microbiome studies previously reported the co-occurrence of *Neisseria* and *Haemophilus* in the salivary ecosystem (De Angelis *et al.*, 2014; Takeshita *et al.*, 2016). A salivary community type comprising *Neisseria*, *Haemophilus*, *Gemella*, *Porphyromonas*, and *Streptococcus mitis* in combination with reduced phylogenetic diversity was associated with better periodontal health (Takeshita *et al.*, 2016). Furthermore, a salivary microbiome study on IBD patients revealed a correlation between elevated salivary IgA levels and a lower abundance of *Neisseria*, *Haemophilus*, *Gemella*, and *Streptococcus* (Said *et al.*, 2014). Salivary IgA levels were previously shown to be elevated in IgAN patients (Yamabe *et al.*, 1987). In the present study, the abundance of the genera *Neisseria*, *Haemophilus*, *Gemella*, and *Streptococcus* increased in IgAN patients, and, thus, was not directly associated with salivary IgA responses in the pathogenesis of IgAN. This may be attributed to differences in the disease pathogeneses of IgAN and IBD. However, since this is the first study to compare the salivary microbiome in IgAN and UC and data on salivary IgA levels in IgAN patients were not collected, further studies are needed to elucidate the relationship between salivary IgA levels and the microbiome in these two diseases.

Some of the OTUs belonging to the genus *Gemella* differentiated the IgAN group from the UC group (Fig. S1B). In contrast to previous findings (Piccolo *et al.*, 2015), this taxa was more abundant in the IgAN group than in the UC and HC groups. These differences in the IgAN salivary microbiome across populations supports the heterogeneity of the IgAN pathology across different ethnicities (Yeo *et al.*, 2018).

*Prevotella* is one of the selected genera that contributes to differentiating IgAN patients from healthy controls. Consistent with previous findings (Piccolo *et al.*, 2015; Cao *et al.*, 2018; Luan *et al.*, 2019), the abundance of *Prevotella* was significantly higher in the HC group than in the IgAN group. A Chinese fecal microbiome study comparing IgAN patients, membranous nephropathy patients, and healthy controls noted a positive correlation between *Prevotella* and a higher serum albumin level. Serum albumin plays an essential role in reducing oxidative stress in mesangial cells by attenuating the production of reactive oxygen species, such as hydrogen peroxide, thereby reducing the risk of IgAN progression towards ESRD (Kawai *et al.*, 2018). Therefore, the genus *Prevotella* appears to play a protective role in the salivary and gut ecosystems, and a decline in its abundance may facilitate systemic disease progression, such as IgAN.

Although the OTU clustering methods performed in the present study were not sufficient for species level assignment, we identified several OTUs assigned to *Prevotella melaninogenica* species, with more than 99% identity, which were among the selected taxa for distinguishing IgAN patients from UC patients, with a significantly higher abundance in UC patients than in IgAN patients. Even though *P. melaninogenica* has been identified as a member of the Japanese salivary core microbiome (Takeshita *et al.*, 2016), previous studies implicated it in the development of oral and systemic diseases (Brook, 1995; Sibley *et al.*, 2011; Kondo *et al.*, 2018). The expression of TLR2 was previously shown to be up-regulated in the blood mononuclear cells of IgAN patients (Chang and Li, 2020; Saito *et al.*, 2016) and in the dendritic cells of IBD patients (CD and UC) (Hug *et al.*, 2018). Since *P. melaninogenica* reportedly stimulates cytokine responses via TLR2 pathways (Kondo *et al.*, 2018), it has the potential to contribute to the progression of inflammatory disorders, such as UC and IgAN. On the other hand, the depletion of CD4+ T cells correlated with an increase in the abundance of *P. melaninogenica* in the salivary microbiota of HIV patients (Lewy *et al.*, 2019). Therefore, the lower abundance of *P. melaninogenica* in IgAN patients in the present study implied the autoimmune nature of IgAN, and further studies are needed to clarify the role of this genus in the T helper cell imbalance in IgAN.

*Streptococcus* is the predominant genus in the phylum *Firmicutes*, and was the most abundant in the CT group, followed by the IgAN group. In previous studies, *Streptococcus* was enriched in the subgingival microbiome of chronic periodontitis patients with IgAN (Cao *et al.*, 2018) and was also identified as one of the core members of the tonsillar crypt microbiome (Jensen *et al.*, 2013). 16S rDNA reads of the *Streptococcaeae* family were found to be elevated in the fecal microbiome of IgAN patients with persistent proteinuria (De Angelis *et al.*, 2014). Another study showed that the level of the cell surface collagen-binding Cnm protein of *S. mutans* was higher in tonsillar specimens from IgAN patients than in those from CT patients (Ito *et al.*, 2019). In the present study, the mean relative abundance of this species was higher in the IgAN group than in the CT group (*P*>0.05). Therefore, some bacterial causal agents of focal tonsillar infection, such as *Streptococcus*, may play a role in the pathogenesis of IgAN because immunological assays of renal tissues from IgAN patients detected streptococcal proteins (Schmitt *et al.*, 2010).

Several OTUs belonging to the genus *Haemophilus* varied significantly between the IgAN and UC groups only. Some members of the genus *Hemophilus*, such as *H. parahaemolyticus*, are commensal microflora in the upper respiratory tract, and may be opportunistic pathogens that cause invasive and severe diseases (Le Floch *et al.*, 2013). *H. parahaemolyticus* belongs to the *H. parainfluenzae* group and is one of the few species in the genus *Haemophilus*, including *H. influenzae*, producing IgA1 protease, which is responsible for its pathogenicity in its human host (Norskov-Lauritsen, 2014). Piccolo *et al.* reported an increase in *H. parainfluenzae* in the salivary microbiota of IgAN patients with the lowest grade of proteinuria (Piccolo *et al.*, 2015). A previous study that recommended the use of *H. influenzae*-derived IgA protease to treat IgAN reported that the lack of mesangial IgA1 specificity by the *H. influenzae*-derived IgA protease limits its immediate application to IgAN therapy (Eitner and Floege, 2008). These findings suggest that some members of the genus *Hemophilus* contribute to the early pathogenic stages of IgAN. Further studies based on these associative members may contribute to a more detailed understanding of the progression of IgAN and, thus, expedite therapeutic applications.

In the present study, the microbial diversity and richness of the salivary microbiome were significantly lower in UC patients than in HC. This was in contrast to previous studies that reported no significant difference in terms of richness or diversity between these two groups (Said *et al.*, 2014; Xun *et al.*, 2018). Furthermore, unlike previous findings (Xun *et al.*, 2018), the *Firmicutes*/*Bacteroidetes* ratio was lower in the UC group than in the HC group. The abundance of *Oribacterium* was significantly lower in UC patients than in HC, which is consistent with previous studies (Said *et al.*, 2014; Xun *et al.*, 2018). While the present study and another study (Xun *et al.*, 2018) reported the enrichment of *Streptococcus* in the salivary microbiome of UC patients, Said *et al.* (2014) showed that it was depleted in UC patients. Moreover, in contrast to previous findings (Said *et al.*, 2014), *Veillonella* was less abundant in the UC group than in the HC group in the present study. We also observed a decrease in the abundance of *Prevotella* in the UC group (though *P*>0.05). This was similar to the findings of the Chinese cohort study (Xun *et al.*, 2018) that showed the significant depletion of *Prevotella* in the UC group. However, Said *et al.* (2014) reported an increase in the abundance of this genus in UC group. These discrepancies may be attributed to multiple factors, such as study design, sample size, and the genetic history of the study subjects.

While some epidemiological studies observed a male predominance among North American and Western European populations (Barratt and Feehally, 2014; Sukcharoen *et al.*, 2020), others showed an equal distribution of the incidence of IgAN between the sexes in an Asian population (Lee *et al.*, 2012; Cheng *et al.*, 2013; Feehally and Barratt, 2015). A single-center study in Japan reported that primary glomerulonephritis was more prevalent in men than in women (Moriyama *et al.*, 2010). In microbiome studies, sex may have acted as a confounding factor because some showed a significant difference between the male and female microbiomes (Raju *et al.*, 2019; Minty *et al.*, 2020). Therefore, upon investigating the existence of a sex-associated microbiota in our cohort, we found that the abundance of some genera significant differed between HC and IgAN males (Fig. S4A), whereas others differed between HC and IgAN females (Fig. S4B). These results suggest that the microbiota plays a role in the sex-associated risk of IgAN disease progression and, thus, further research in this field is needed.

There were some limitations that need to be addressed. The present study included a small and non-uniform sample size across the groups. The imbalance in the dataset was not significant and did not affect the microbiome analysis. Disease predictive modeling is affected by an imbalanced dataset; however, the RF classifier is relatively robust when dealing with an imbalanced dataset (Dittman *et al.*, 2015) and the AUC-RF package uses a RF classifier for modeling. Furthermore, data associated with the stage of disease for IgAN patients was not available for a clinical metadata correlation analysis. However, since tonsillectomy is recommended and effective in the early stages of IgAN (Hotta *et al.*, 2001) and IgAN patients in the present study provided saliva samples before tonsillectomy, these patients were in the early stage of the disease. Moreover, our study design is limited by the lack of clinical data reporting serum or salivary IgA levels in IgAN patients. Since secretory IgA plays an important role in the pathogenesis of IgAN, a future microbiome study that investigates the relationship between secretory IgA levels and the predominant microbiota associated with IgAN is warranted.

In conclusion, the results of the present study, which examined variations in the salivary microbiome profiles of IgAN patients, UC patients, CT patients, and HC, indicate the potential of the salivary microbiome as a biomarker source for the development of a non-invasive diagnostic tool for IgAN. However, the biological role of the microbial biomarkers identified in this study in the pathogenesis and disease progression of IgAN is a scope for further research.

## Citation

Khasnobish, A., Takayasu, L., Watanabe, K., Nguyen, T. T. T., Arakawa, K., Hotta, O., et al. (2021) Dysbiosis in the Salivary Microbiome Associated with IgA Nephropathy—‍A‍ ‍Japanese Cohort Study. *Microbes Environ ***36**: ME21006.

https://doi.org/10.1264/jsme2.ME21006

## Supplementary Material

Supplementary Material 1

Supplementary Material 2

## Figures and Tables

**Table 1.** Subject demographics in four different groups—IgAN, CT, UC, and HC.

Demographics	IgAN (*n*=43)	CT (*n*=20)	UC (*n*=22)	HC (*n*=50)
Age, years, median (IQR)	39 (20.5)	34.5 (14.5)	44.5 (7.75)	37.5 (8)
Male	20	13	11	36
Female	23	7	11	14

Median age in terms of years is shown for each group along with IQR in parentheses. Small IQR values represent data points that are spread closer to the median. IgAN, Immunoglobulin A nephropathy; CT, chronic tonsillitis; UC, ulcerative colitis; HC, healthy control; IQR, interquartile range.

**Table 2.** Permutational multivariate analysis of variance (PERMANOVA) in salivary microbiome samples among four groups—IgAN, CT, UC, and HC.

Category	No. of Subjects	Weighted UniFrac			Unweighted UniFrac	
		R^2^	Adjusted *P* value		R^2^	Adjusted *P* value
CT vs HC	CT: 20 HC: 50	0.06	**0.006**		0.06	**0.001**
IgAN vs CT	IgAN: 43 CT: 20	0.02	0.217		0.03	**0.003**
IgAN vs HC	IgAN: 43 HC: 50	0.07	**0.003**		0.03	**0.001**
CT vs UC	CT: 20 UC: 22	0.04	0.179		0.04	**0.002**
UC vs HC	UC: 22 HC: 50	0.04	**0.043**		0.05	**0.001**
IgAN vs UC	IgAN: 43 UC: 22	0.06	**0.003**		0.04	**0.001**

*P* values were adjusted for multiple testing by the Benjamin-Hochberg method. *P* values <0.05 are in bold.

**Table 3.** Mean area under curve for salivary microbiome samples among IgAN, CT, UC, and HC groups.

Category	cvAUC	
	OTU Level	Genus Level
IgAN vs HC	0.88	0.90
IgAN vs CT	0.62	0.707
IgAN vs UC	0.88	0.851

The mean AUCs (cvAUC) of the 10-fold cross validation process repeated 20 times using the best RF model in the AUC-RF package are shown here. cvAUC, mean area under curve from 20 repetitive 10-fold cross validations of the random forest model; RF, Random Forest.
