# Supplementary material for: Dysbiosis in the Salivary Microbiome Associated with IgA Nephropathy—‍A‍ ‍Japanese Cohort Study
Source: Microbes Environ. 2021 Jun 1;36(2):ME21006. doi: 10.1264/jsme2.ME21006 (PMC8209455; doi:10.1264/jsme2.ME21006)
Supplement: Supplementary file 1 — Supplementary Material 1 [file 36_21006_s1.pdf]

Supplementary materials

Fig. S1. Biomarkers of IgAN compared to CT, UC, and HC groups. Histogram of LDA score indicating the effective size and ranking of each differentially abundant OTU is shown here for (A) HC vs IgAN; (B) IgAN vs UC; and (C)IgAN vs CT. LDA>2 threshold was used for LEfSe analysis. LEfSe, Linear discriminant analysis (LDA) Effect Size; IgAN, Immunoglobulin A Nephropathy; CT, Chronic Tonsillitis; UC, Ulcerative Colitis; HC, Healthy Control; OTU, operational taxonomic unit.

Fig. S2. Genus level variables from Random Forest (RF) analysis of the salivary microbiota using AUC-RF package. The mean relative abundance of taxa selected by AUC-RF are shown here.(A)Six unique genera selected for IgAN vs HC model; (B) One unique genera selected for IgAN vs CT model; and (C)Common genera selected for IgAN vs HC and IgAN vs CT model . * p-value <0.05; ** p-value < 0.01; *** p-value <0.001 based on Wilcox test with Benjamin-Hochberg correction. IgAN=blue, CT=red, and HC=purple. IgAN, Immunoglobulin A Nephropathy; CT, Chronic Tonsillitis; HC, Healthy Control; OTU, operational taxonomic unit.

Fig. S3. Alpha and beta diversity in IgAN male, HC male, IgAN female and HC female subjects. Samples from 20 IgAN male, 36 HC male, 23 IgAN female and 14 HC female subjects are shown. (A) The observed and Chao1-estimated OTU numbers, and the Shannon index of salivary microbiome from the four groups. * p-value <0.05; ** p-value < 0.01; *** p-value <0.001 based on Wilcox test. (B) Unweighted UniFrac –PCoA and (C) weighted UniFrac –PCoA of the salivary microbiome from the four groups (IgAN=blue, and HC=purple). IgAN, Immunoglobulin A Nephropathy; CT, Chronic Tonsillitis; UC, Ulcerative Colitis; HC, Healthy Control; OTU, operational taxonomic unit; PCoA, principal coordinate analysis.

Fig. S4. Unique Gender-associated genera with significant difference between the IgAN and HC groups. (A) 12 genera with more than 0.1% mean relative abundance and p-value < 0.05 between IgAN male and HC male groups are shown. (B) 5 genera with more than 0.1% mean relative abundance and p-value <0.05 between IgAN Female and HC male are shown.

Fig. S1 | Anushka Khasnobish

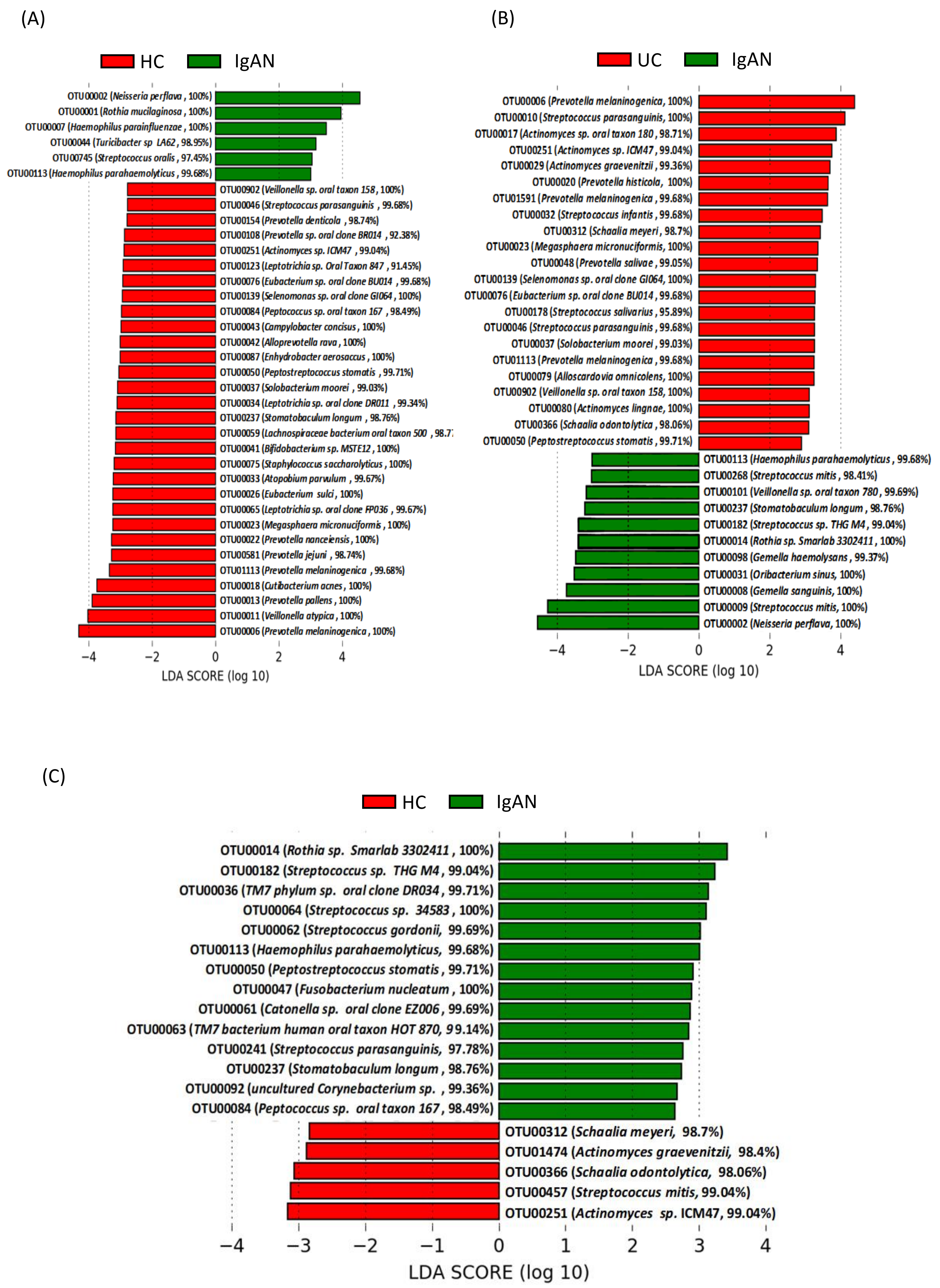

Fig. S2 | Anushka Khasnobish

(A)

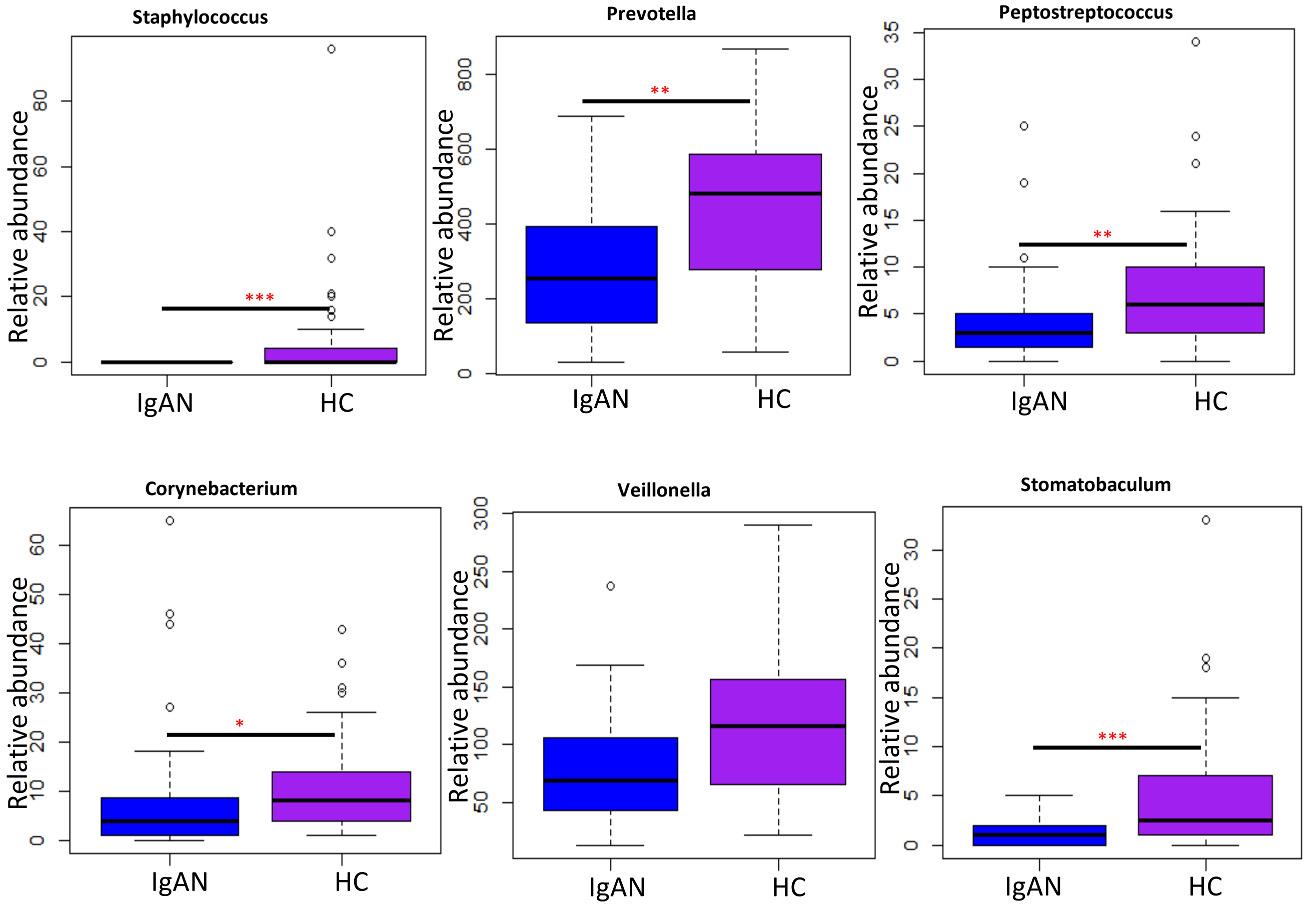

(B)

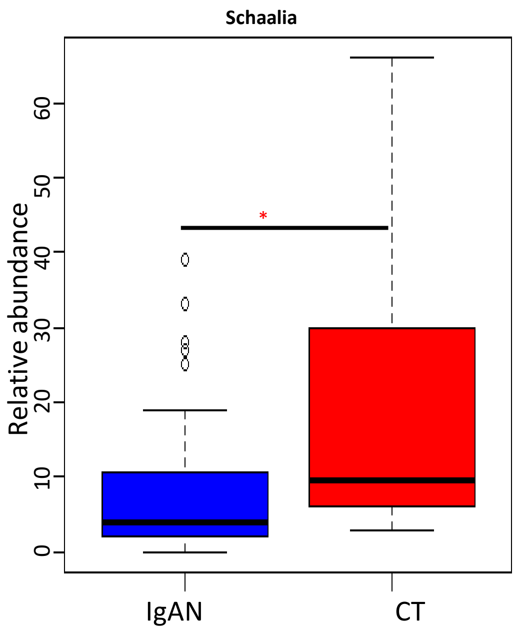

(C)

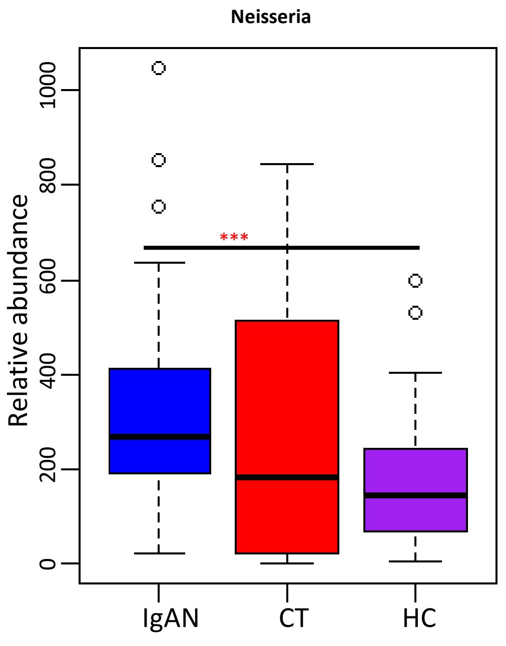

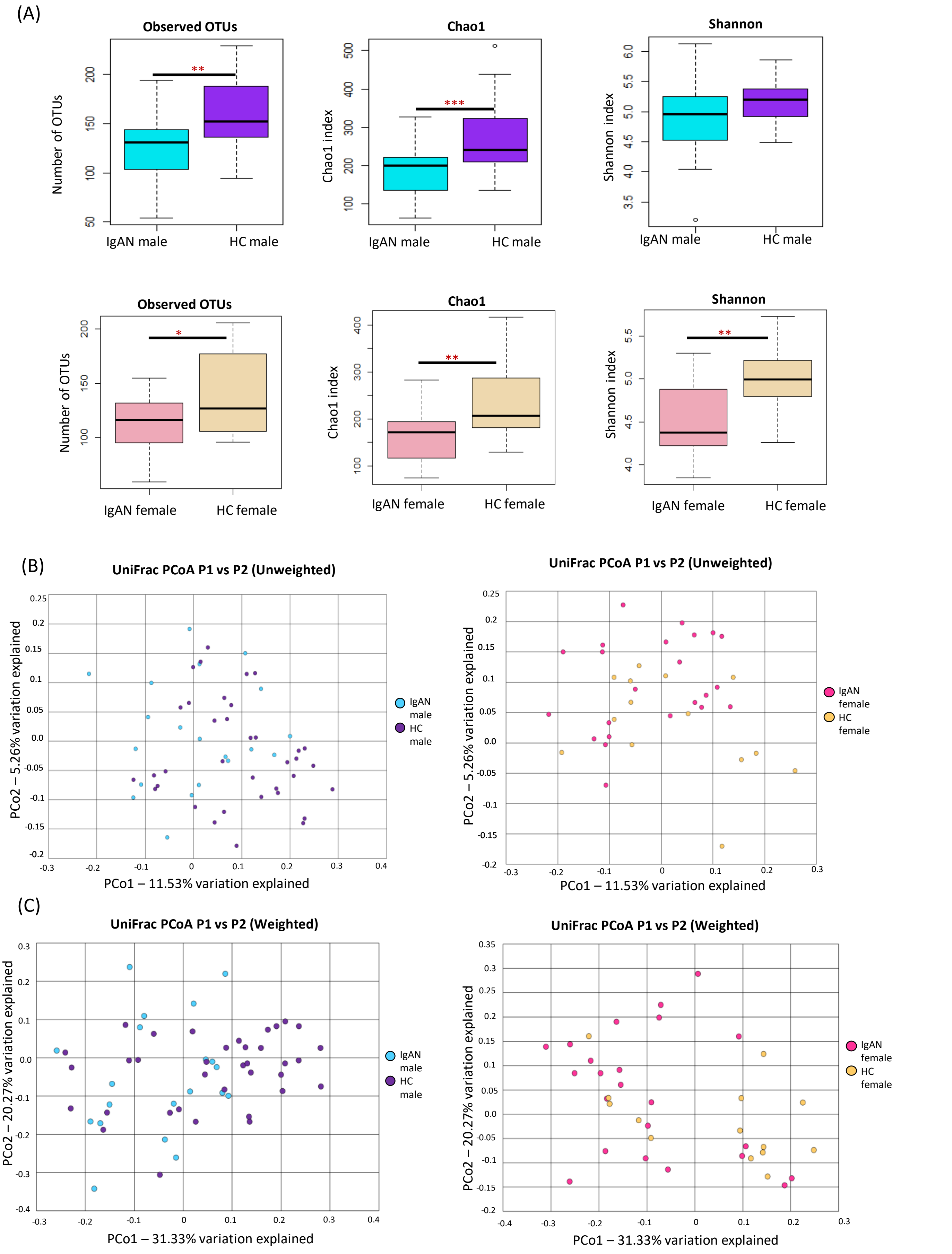
(A)
Observed OTUs
Number of OTUs
**
IgAN male
HC male
Chao1
Chao1 index
***
IgAN male
HC male
Shannon
Shannon index
IgAN male
HC male
Observed OTUs
Number of OTUs
*
IgAN female
HC female
Chao1
Chao1 index
**
IgAN female
HC female
Shannon
Shannon index
**
IgAN female
HC female
(B)
UniFrac PCoA P1 vs P2 (Unweighted)
PCo2 – 5.26% variation explained
PCo1 – 11.53% variation explained
IgAN male
HC male
UniFrac PCoA P1 vs P2 (Unweighted)
PCo2 – 5.26% variation explained
PCo1 – 11.53% variation explained
IgAN female
HC female
(C)
UniFrac PCoA P1 vs P2 (Weighted)
PCo2 – 20.27% variation explained
PCo1 – 31.33% variation explained
IgAN male
HC male
UniFrac PCoA P1 vs P2 (Weighted)
PCo2 – 20.27% variation explained
PCo1 – 31.33% variation explained
IgAN female
HC female

(A)

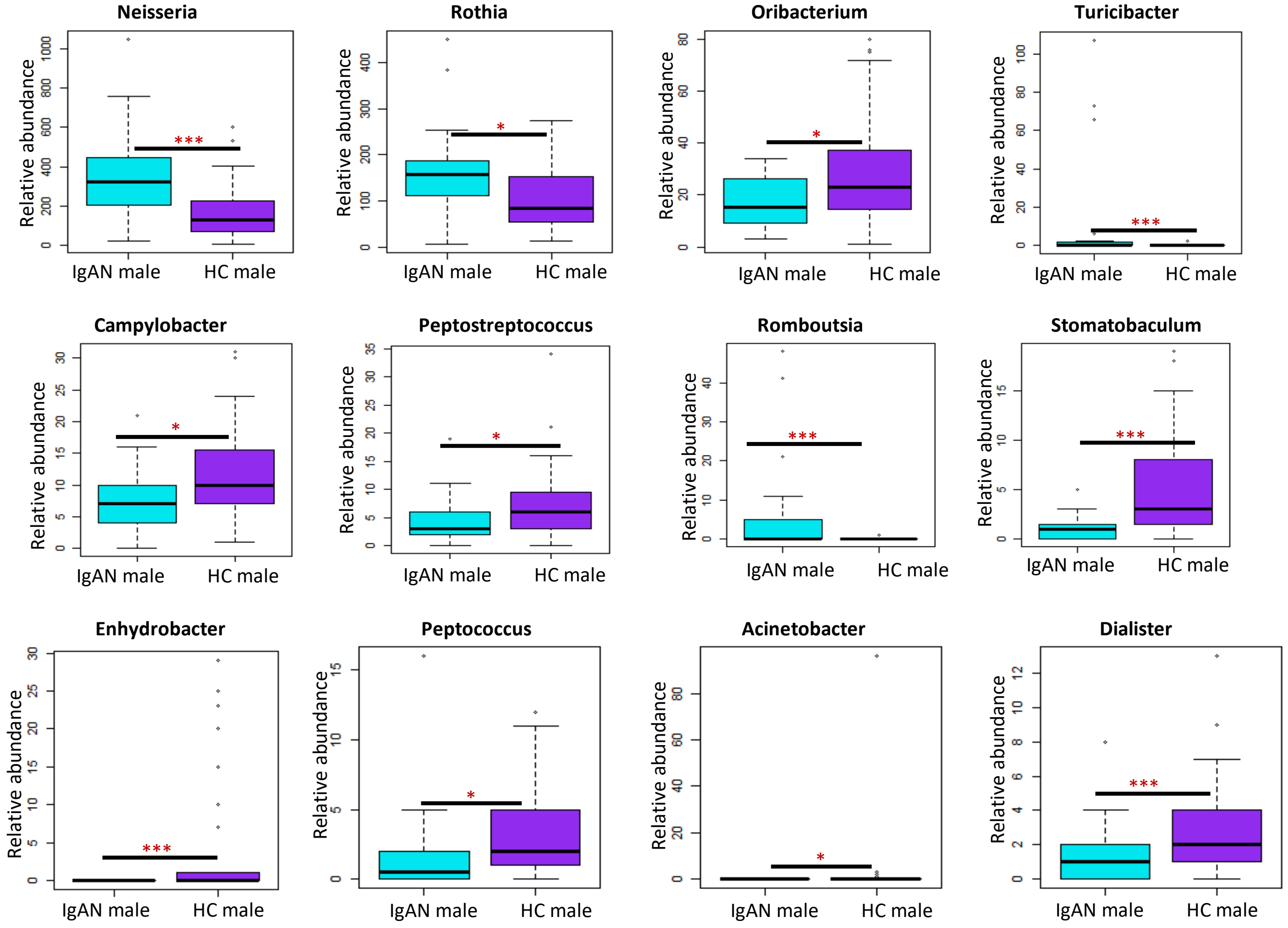
Neisseria
Rothia
Oribacterium
Turicibacter
Campylobacter
Peptostreptococcus
Romboutsia
Stomatobaculum
Enhydrobacter
Peptococcus
Acinetobacter
Dialister
Relative abundance
IgAN male
HC male
***
*

(B)

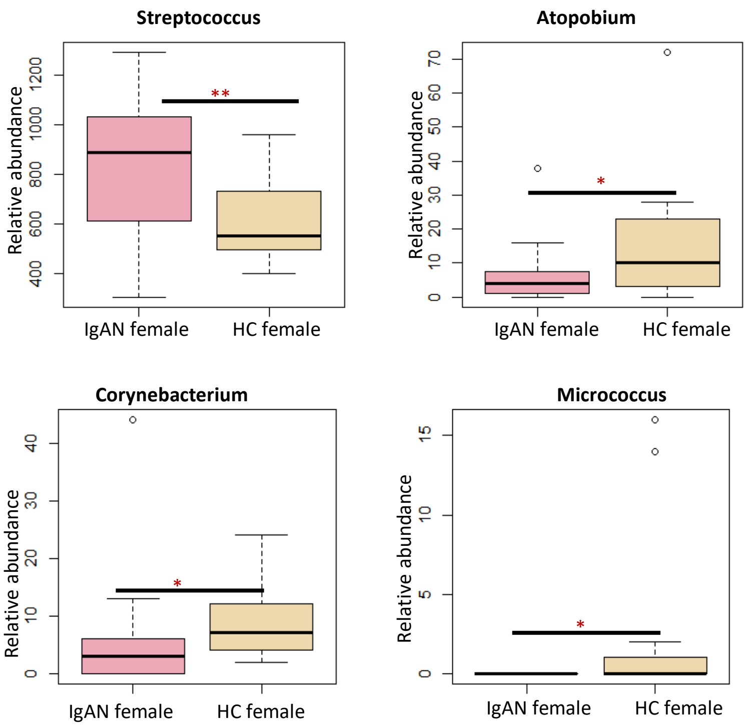
Streptococcus
Atopobium
Corynebacterium
Micrococcus
Relative abundance
IgAN female
HC female
**
*

Table S1. Statistics of 16S V1-V2 pyrosequencing of salivary microbiome samples

Total Reads	Filter-Passed Reads	Reads Removed		
		Reads lacking primer sequences	Reads with average Qv<25	Possible chimeric reads
1576683 (100)	809607 (51.3)	758604(48.1)	3683(0.2)	10478(0.01)

Note: The number in the parentheses indicate the ratio in percentage to the total read number.

Table S3. Permutational multivariate analysis of variance (PERMANOVA) in salivary microbiome samples in the four groups to check gender effect. The adjusted p-values were adjusted for multiple testing by Benjamin-Hochberg method. p-value <0.05 are in bold.

Category	No. of subjects	Weighted UniFrac		Unweighted UniFrac	
		R^2	p-value	R^2	p-value
CT(male vs female)	male:13<br>female:7	0.12155	0.06194	0.05878	0.2198
**HC(male vs female)**	**male:36**<br>**female:14**	0.01044	0.8362	0.03011	**0.04196**
UC(male vs female)	male:11<br>female:11	0.05306	0.3087	0.04002	0.7413
**IgAN(male vs female)**	**male:20**<br>**female:23**	0.05431	**0.03696**	0.02829	0.1748
**All(male vs female)**	**male:80**<br>**female:55**	0.02927	**0.003996**	0.01096	**0.02697**

Table S4. Permutational multivariate analysis of variance (PERMANOVA) in salivary microbiome samples in the two groups, namely IgAN and HC, which showed within group gender effect. The adjusted p-values were adjusted for multiple testing by Benjamin-Hochberg method. p-value <0.05 are in bold.

Category	No. of subjects	Weighted UniFrac			Unweighted UniFrac		
		R^2	p-value	Adjusted p-value	R^2	p-value	Adjusted p-value
**IgAN female vs HC female**	HC:14<br>IgAN:23	0.08802	**0.01598**	**0.01598**	0.0499	**0.001998**	**0.002**
**IgAN male vs HC male**	HC:36<br>IgAN:20	0.06467	**0.004995**	**0.00999**	0.04499	**0.000999**	**0.002**
